# Socioeconomic and Demographic Effects on SARS-CoV-2 Testing: Evidence From the State of Uttar Pradesh, India

**DOI:** 10.7759/cureus.59521

**Published:** 2024-05-02

**Authors:** Raghukul R Pandey, Monika Agarwal, Brian P Wahl, Tushar Garg, Amita Jain

**Affiliations:** 1 Microbiology, King George's Medical University, Lucknow, IND; 2 Community Medicine and Public Health, King George's Medical University, Lucknow, IND; 3 International Health, Johns Hopkins Bloomberg School of Public Health, Baltimore, USA

**Keywords:** india, uttar pradesh, demographic factors, socioeconomic factors, utilisation, access, testing, sars-cov-2, covid-19

## Abstract

Background

The rapid global spread of SARS-CoV-2 highlighted critical challenges in healthcare systems worldwide, with differences in testing access and utilization becoming particularly evident. This study investigates the socioeconomic and demographic factors influencing SARS-CoV-2 testing service access and utilization during the second wave of the pandemic in Uttar Pradesh (UP), India.

Methods

The study was conducted from July to October 2023 in two districts of Uttar Pradesh (UP). These districts were chosen because one had the highest and the other the lowest SARS-CoV-2 testing rates per million population as reported from March to June 2021. The study population included consenting adult individuals with self-reported symptoms indicative of SARS-CoV-2 infection during March-June 2021. The study excluded individuals under 18 years, those who did not consent, pregnant or lactating mothers, and those with communication-impairing medical conditions. Data were collected using a structured questionnaire based on Andersen's Behavioural Model of Health Services Use. We used chi-squared tests for all categorical variables to obtain p-values and Poisson regression to identify factors influencing testing rates.

Results

We screened 4,595 individuals and identified 675 eligible participants for this study. Adjusted prevalence ratios derived from multiple variate Poisson regression models showed that participants in Sitapur had a 0.47 (95% CI: 0.39-0.57) times the prevalence of being tested than those in Lucknow. Furthermore, individuals from other backward castes and scheduled castes had a 1.15 (95% CI: 0.99-1.34) and 1.22 (95% CI: 0.95-1.56) times prevalence of being tested for SARS-CoV-2, respectively, when compared to the general caste population. Scheduled Tribes showed a higher prevalence of being tested, contrasting with existing literature. Households with low, middle, and high income showed a 1.46 (95% CI: 1.12-1.89), 1.52 (95% CI: 1.14-2.02), and 1.73 (95% CI: 1.23-2.45) times the prevalence of SARS-CoV-2 testing compared to those below the poverty line, respectively. Behavioral factors such as media use showed an inverse relationship with testing prevalence; individuals who did not watch TV at all had a 0.83 (95% CI: 0.70-0.99) times prevalence of being tested compared to frequent viewers, and similarly, those not using the internet on mobiles had a 0.82 (95% CI: 0.67-0.99) times prevalence than daily users. Individuals using private healthcare facilities had a 0.87 (95% CI: 0.77-0.99) times prevalence of SARS-CoV-2 testing compared to those using government facilities.

Conclusions

These findings highlight the importance of public health strategies that address socio-economic and behavioral disparities to ensure equitable testing access across all community groups.

## Introduction

The COVID-19 pandemic placed unprecedented stress on health systems with demand for diagnostics and treatment services often exceeding availability even in the most well-resourced settings [1,2]. Large-scale diagnostic testing is a key tool in epidemiology and was crucial in containing the COVID-19 pandemic [3]. Testing for SARS-CoV-2 was typically performed for one of the two reasons. First, a symptomatic patient might be tested to inform their clinical treatment. Second, testing might be performed to screen infectious individuals with a focus on public health outcomes [4]. “Test, test, test” was the key to controlling the spread of SARS-CoV-2 and its clinical manifestation in the initial phase of the COVID-19 pandemic [5]. It rapidly gained traction as an intervention that could avoid both the immediate economic costs of lockdown and the societal costs of social distancing measures. However, technical, regulatory, and logistical challenges obstructed the response to this testing approach [6]. These challenges were further exacerbated by disparities in healthcare access and utilization, largely influenced by health beliefs and socioeconomic and demographic factors [4].

The decision to seek SARS-CoV-2 testing was influenced by factors such as health literacy, access to information, current health status, and socioeconomic considerations, all of which become more complex during a pandemic. Awareness of the pandemic and its impact, and knowledge of the logistics around testing are crucial. Health status plays a significant role, with those experiencing mild symptoms often avoiding testing, while individuals with severe symptoms face challenges accessing testing facilities. Financial burdens and systemic inefficiencies, such as delayed responses and shortages of resources, present barriers, particularly in economically disadvantaged areas and places with poor health infrastructure. These challenges not only discouraged testing but also contributed to the underreporting of COVID-19 cases, especially in the early stages of the pandemic, highlighting disparities in access to healthcare and the importance of addressing these systemic issues to ensure equitable health outcomes [7,8].

In India, response to SARS-CoV-2 testing was hindered by limited healthcare infrastructure at the initial stage. Inequitable access further worsened the situation, with significant disparities in testing availability between urban and rural regions, and among different socio-economic groups. Misinformation and lack of awareness about testing protocols, alongside some financial barriers, discouraged individuals from seeking tests. Socioeconomic factors strongly influence healthcare access and utilization, encompassing income, education, insurance, age, gender, religion, caste, and location [9]. Socio-economic and demographic diversity of India presents unique challenges in public health service delivery and there is a need to understand how these dynamics influenced testing service utilization during a crisis in Indian contexts.

When the first case of COVID-19 was reported in Uttar Pradesh (UP) on March 3, 2020, King George’s Medical University, Lucknow was the only lab in the state able to conduct only 60 RT-PCR tests per day to diagnose SARS-CoV-2. Just before the second epidemic wave in March 2021, UP expanded its testing capacity to over 150,000 tests daily across 236 laboratories, a mix of 126 government and 110 private sector facilities. The state experienced the second epidemic wave of COVID-19 between March and June 2021, which was acute and overwhelmed the state's health system. The intensity of the second wave was attributed to the emergence of the highly transmissible variant of concern B.1.617.2, also known as the Delta variant, which was first sequenced in Maharashtra in late 2020 [10-12].

Prior studies have broadly addressed the efficacy of testing on national and global scales [13]. Preliminary observations indicated significant disparities in SARS-CoV-2 testing access and utilization across different districts of UP, hinting at underlying socioeconomic and demographic influences. Our study focuses on a state with unique socioeconomic and demographic characteristics that influence health service utilization, particularly SARS-CoV-2 testing. There is a lack of comprehensive research examining the interplay of socioeconomic and demographic factors in healthcare access during pandemics in India. Our study examines these factors, which have implications for public health strategies and policy-making [14]. It also provides a detailed examination of the disparities in testing access and utilization between urban and rural areas during the acute phase of a pandemic, a dynamic not extensively documented in existing literature [15].

Although the acute phase of the COVID-19 pandemic may have passed, the lessons learned remain critical for preparing for future healthcare crises. The pandemic has also underscored the importance of equitable healthcare access. The findings of our study can guide policymakers and public health officials in structuring targeted interventions to improve testing rates and accessibility, especially in regions similar to UP that may face healthcare infrastructure challenges.

## Materials and methods

Study setting

Uttar Pradesh (UP), situated in northern India, presents a unique study setting due to its demographic size and health system challenges. It is the most populous state of India with over 232 million population. It is mostly rural (78% population) with more than 40% people under the age of 20 years and a literacy rate of 68% [16]. Key health system challenges in the state include inadequate healthcare infrastructure, shortage of medical personnel, limited access to quality healthcare in rural areas, and high patient load on existing facilities.

UP developed a dedicated COVID-19 portal during the initial stage of the pandemic. This portal contained near-complete coverage of SARS-CoV-2 testing activities due to mandatory reporting protocols for public and private sector laboratories. We selected two districts based on the test per million population (TPM) during March-June 2021 - Sitapur with the lowest TPM rate at 53,723 TPM and Lucknow with the highest at 385,673 TPM [17]. District Lucknow, as the state capital, has a better healthcare infrastructure, and 66.18% of its population resides in urban areas. Conversely, Sitapur represents a largely rural setting with 88.16% of its population living in rural areas and having relatively limited healthcare resources [16].

Study design

We conducted a cross-sectional study from July to October 2023 in two districts of Uttar Pradesh (UP) that reported the maximum and minimum SARS-CoV-2 tests per million population during the second wave of the COVID-19 pandemic. The second wave in UP occurred between March and June 2021. This period was selected to assess the impact of the acute phase on testing demand and access in the contrasting urban and rural settings of the Lucknow and Sitapur districts.

Study population

Our study included adults aged 18 years or older who resided in Lucknow or Sitapur district. To qualify for the study, participants had to have experienced three pre-identified symptoms indicative of SARS-CoV-2 infection simultaneously between March and June 2021. These symptoms included: a new fever or a feeling of feverishness accompanied by chills or sweating, a cough, and mild or moderate difficulty in breathing, which could involve breathing faster than normal, difficulty fully inhaling or exhaling, or wheezing on exhalation. Additionally, participants were required to provide informed consent, be physically and mentally capable of responding to our questions, and understand Hindi. Exclusion criteria included individuals under 18 years old, non-consenting individuals, pregnant or lactating mothers, anyone with a medical condition that might hinder effective communication, and residents of districts other than Lucknow or Sitapur.

Sample design

The calculation of the sample size for this study was guided by the principle of Event Per Variable (EPV), specifically tailored to regression models with binary outcomes [18]. Adhering to this principle, we used the following formula for determining the sample size:

 Sample size(n)=100+x×i

Here, x represents a predetermined integer value, and i denotes the number of independent variables planned for inclusion in the final regression model. To arrive at a reasonable sample size, x was fixed at 50, making the EPV formula effectively n=100+50i. Anticipating the inclusion of at least 10 independent variables into the final model, the computed sample size was:

 Sample size(n)=100+50×10=600

Factoring in a 10% non-response rate to accommodate potential dropouts or non-participation, the requisite sample size was adjusted to approximately 660. We adopted a multistage cluster sampling technique to select study participants. After selecting the highest and lowest TPM districts, we further identified community development blocks (CDB) and urban wards with the highest and lowest TPM within each district. Through this approach, we selected four CDBs and four urban wards from both districts. Then, we identified primary sampling units (PSUs) from a list of census villages and enumeration blocks (EBs) in the selected CDBs and urban wards, respectively. The selection of the required number of villages and EBs was proportional to the urban and rural population distributions of districts. We choose PSUs based on probability proportional to size (PPS) criteria, using the Census 2011 data as the sampling frame.

We further segmented each PSU into four equal quadrants after dividing the total number of households (HHs) in each village or EB, a process validated with the help of residents. We systematically selected five HHs reporting eligible individuals from each quadrant through a circular random sampling method to select around 20 HHs per village or EB. We selected one consenting Hindi-speaking eligible adult from each selected household. In cases where multiple eligible individuals lived in a single household, we used the KISH table method to select the respondent [19]. We also tried to choose female respondents in every alternate household to maintain gender balance.

Data collection

We used Andersen's Behavioral Model of Health Services Use to design a structured questionnaire for data collection. This model considers access to healthcare as a result of predisposing factors (demographic and social), enabling/disabling factors (economic, knowledge), and need factors (health outcome) [20]. Initially, we developed this questionnaire in English and ensured the inclusion of relevant questions that aligned with the objectives of the study. The questionnaire was later translated and administered in Hindi, the local language of the region. Before the study, we pretested the questionnaire involving 33 respondents in an unrelated population. We collected the data between July and October 2023 from all eligible participants who provided their consent using digital tablets for mobile data collection.

Data analysis

Data processing and statistical analyses were performed using Stata version 18 (StataCorp LLC, College Station, TX, USA). A series of categorical variables, including age category, gender, religion, marital status, education, average monthly individual income in 2021, newspaper readership, mobile internet usage, and tobacco use were compared across two districts of Lucknow and Sitapur using the chi-square test for independence. Fisher’s exact test was used for caste, occupation before the COVID-19 pandemic, average household monthly income in 2021, household size, watching TV, and usual healthcare source in the last five years where the expected values in any of the cells of the table were below 5.

Subsequently, we modeled prevalence ratios using univariate and multivariate Poisson regression to compare being tested for SARS-CoV-2 with not being tested [21]. In this analysis, the ratio represents the prevalence of individuals undergoing SARS-CoV-2 testing based on various factors, including district residence, age, gender, religion, caste, marital status, education, occupational status before and after the COVID-19 pandemic, average individual and household monthly income, household size, and behavioral factors such as media use and healthcare sources. It also quantifies how many times more (or less) likely individuals with certain characteristics (like living in a particular district or belonging to a specific socioeconomic group) are to be tested for SARS-CoV-2 compared to a reference group.

Human participation protection

We obtained approval from the Institutional Human Ethics Committee of King George’s Medical University, Lucknow, UP to conduct this study (Ref. Code: 119th ECM II B-Ph.D/P1). We gained written informed consent from all the participants before conducting the questionnaire.

## Results

Study population characteristics

We screened 4,595 individuals across Lucknow and Sitapur, identifying 675 eligible participants, with a nearly equal distribution between these two districts. A significant rural-urban divide was observed (p < 0.001), with more urban participants in Lucknow. Age, gender, and religious connections showed no significant differences across districts. However, caste composition varied significantly (p < 0.001), with Lucknow having more General caste individuals and Sitapur more from Other Backward Castes.

As per Table 1, educational levels and occupational backgrounds differed between the districts (p < 0.001), with higher education and regular salaried jobs more common in Lucknow, while Sitapur had more individuals with no education and in homemaking or self-employment. There was no significant difference in occupational changes due to the COVID-19 pandemic (p = 0.305).

**Table 1:** Demographic and socioeconomic characteristics of study participants in Lucknow and Sitapur districts * p-values are derived from chi-squared tests for age category, gender, religion, marital status, education, change in occupation after the COVID-19 pandemic, average individual monthly income in 2021, using the Internet on a mobile and tobacco use in the last three years. ** p-values are derived from Fisher’s exact tests for caste, occupation before the COVID-19 pandemic, average household monthly income in 2021, household size, watching TV and usual healthcare source in the last five years.

Characteristics	District			p-value
	Lucknow	Sitapur	Total	
Age category*				
18-29	79	74	153	0.056
30-39	77	102	179	
40-49	75	87	162	
50-59	58	55	113	
60+	43	25	68	
Gender*				
Female	160	177	337	0.376
Male	172	166	338	
Religion*				
Hindu	310	313	623	0.102
Muslim	18	30	48	
Caste**				
General	66	106	172	<0.001
Other backward caste	230	165	395	
Scheduled caste	36	71	107	
Scheduled tribe	0	1	1	
Marital status*				
Never married	49	37	86	0.290
Married	269	291	560	
Widowed	12	14	26	
Education*				
No education	56	107	163	<0.001
Primary education	22	46	68	
Secondary education	117	146	263	
Graduation	93	33	126	
Postgraduation	44	11	55	
Occupation before COVID-19 pandemic**				
Homemaker	108	142	250	<0.001
Regular salaried	72	24	96	
Self‐employed	63	96	159	
Student	34	10	44	
Labour	53	61	114	
Unpaid worker	1	10	11	
Change in occupation after COVID-19 pandemic*				
Lost	6	12	18	0.305
Lost but regained	57	51	108	
No Change	269	280	549	
Average individual monthly Income 2021*				
No income	146	169	315	<0.001
Below Poverty Line	34	91	125	
Low income	84	71	155	
Middle income	67	12	79	
Average household monthly Income 2021**				
Below Poverty Line	38	68	106	<0.001
Low income	138	207	345	
Middle income	143	65	208	
High income	13	2	15	
Household size**				
1	1	3	4	<0.001
2	17	17	34	
3	35	19	54	
4	80	37	117	
5	104	95	199	
>5	95	172	267	
Watching TV**				
Almost every day	249	142	391	<0.001
Not at all	75	201	276	
Sometimes	8	0	8	
Using the Internet on a mobile*				
Almost every day	256	118	374	<0.001
Not at all	75	225	300	
Usual healthcare source in last 5 years**				
Government facility	149	123	272	<0.001
Private facility	183	205	388	
Traditional healers	0	15	15	
Tobacco use in the last 3 years*				
No	264	205	469	<0.001
Yes	67	138	205	

Income levels and household sizes also showed significant differences (p < 0.001), with Sitapur reporting lower incomes and larger household sizes. TV watching and internet usage were significantly higher in Lucknow (p < 0.001). Healthcare preferences over the last five years differed significantly, with a preference for private facilities in both districts.

Factors affecting SARS-CoV-2 testing

As per Table 2, Sitapur residents showed a significantly lower adjusted Prevalence Ratio (PR) of 0.47 (95% CI: 0.39, 0.57) for testing compared to Lucknow. Age and gender were not significant factors in the adjusted analysis, indicating their minimal role in determining testing rates. Religious connection showed no significant impact on testing rates. However, caste did influence outcomes, with Other Backward Castes and particularly Scheduled Tribes showing a significantly higher adjusted PR of 5.33 (95% CI: 3.54, 8.02).

**Table 2:** Association of demographic and socioeconomic factors with SARS-CoV-2 testing in two districts of Uttar Pradesh, India. * Unadjusted prevalence ratios are derived from bivariate Poisson regression models with whether the individual was tested for SARS-CoV-2 or not. ** Adjusted prevalence ratios are derived from multiple variate Poisson regression models adjusting for district, age category, gender, religion, caste, marital status, education, occupation before the COVID-19 pandemic, occupation change after the first wave of the COVID-19 pandemic, average individual monthly income in 2021, average household monthly income in 2021, household size, watch TV, use of the Internet on mobile, usual healthcare source in last 5 years, and use of tobacco in last 3 years.

SARS-Cov-2 tested	Unadjusted*		Adjusted**	
	PR	95% CI	PR	95% CI
District (Ref: Lucknow)				
Sitapur	0.40	(0.35, 0.47)	0.47	(0.39, 0.57)
Age category (Ref: 18-29 years)				
30-39	0.91	(0.75, 1.11)	0.90	(0.74, 1.11)
40-49	1.05	(0.88, 1.26)	1.02	(0.83, 1.25)
50-59	1.05	(0.86, 1.28)	1.01	(0.82, 1.24)
60+	1.16	(0.94, 1.44)	1.01	(0.80, 1.27)
Gender (Ref: Female)				
Male	1.15	(1.01, 1.30)	0.94	(0.79, 1.12)
Religion (Ref: Hindu)				
Muslim	0.99	(0.77, 1.27)	1.09	(0.87, 1.37)
Caste (Ref: General)				
Other backward caste	1.19	(1.02, 1.40)	1.15	(0.99, 1.34)
Scheduled caste	0.93	(0.73, 1.17)	1.22	(0.95, 1.56)
Scheduled tribe	1.87	(1.63, 2.15)	5.33	(3.54, 8.02)
Marital status (Ref: Married)				
Never married	0.95	(0.78, 1.16)	0.82	(0.62, 1.08)
Widowed	0.77	(0.51, 1.18)	0.85	(0.58, 1.25)
Education (Ref: No education)				
Primary education	1.12	(0.83, 1.50)	1.02	(0.77, 1.35)
Secondary education	1.25	(1.02, 1.53)	0.89	(0.71, 1.11)
Graduation	1.68	(1.38, 2.05)	0.93	(0.72, 1.21)
Postgraduation	1.99	(1.64, 2.42)	1.04	(0.79, 1.35)
Occupation before the COVID-19 pandemic (Ref: Homemaker)				
Unpaid worker	1.07	(0.61, 1.85)	2.26	(0.98, 5.21)
Labour	1.13	(0.93, 1.38)	1.31	(0.65, 2.65)
Student	1.20	(0.92, 1.56)	1.22	(0.89, 1.66)
Self‐employed	1.08	(0.90, 1.30)	1.43	(0.71, 2.89)
Regular salaried	1.69	(1.46, 1.95)	1.69	(0.83, 3.44)
Occupation change after the first wave of the COVID-19 pandemic (Ref: Lost)				
Lost but regained	0.94	(0.68, 1.28)	0.94	(0.45, 1.95)
No Change	0.79	(0.59, 1.06)	0.78	(0.38, 1.58)
Average individual monthly income in 2021 (ref: No income)				
Below Poverty Line	0.79	(0.63, 0.99	0.67	(0.33, 1.34)
Low income	1.29	(1.11, 1.49)	0.78	(0.39, 1.55)
Middle income	1.63	(1.43, 1.86)	0.68	(0.33, 1.39)
Average household monthly income in 2021 (Ref: Below Poverty Line)				
Low income	1.50	(1.15, 1.97)	1.46	(1.12, 1.89)
Middle income	2.01	(1.55, 2.62)	1.52	(1.14, 2.02)
High income	2.54	(1.91, 3.37)	1.73	(1.23, 2.45)
Household size (Ref: 1)				
2	1.29	(0.47, 3.56)	0.78	(0.29, 2.10)
3	1.33	(0.49, 3.62)	0.71	(0.26, 1.92)
4	1.47	(0.55, 3.94)	0.76	(0.28, 2.04)
5	1.11	(0.41, 2.97)	0.69	(0.26, 1.86)
>5	1.06	(0.40, 2.85)	0.77	(0.29, 2.06)
Watch TV (Ref: Almost every day)				
Not at all	0.56	(0.48, 0.65)	0.83	(0.70, 0.99)
Use of the Internet on mobile (Ref: Almost every day)				
Not at all	0.54	(0.47, 0.63)	0.82	(0.67, 0.99)
Usual healthcare source in last 5 years (Ref: Government facility)				
Private facility	0.88	(0.78, 1.00)	0.87	(0.77, 0.99)
Traditional healers	0.52	(0.25, 1.07)	1.03	(0.51, 2.08)
Use of tobacco in last 3 years (Ref: No)				
Yes	0.87	(0.75, 1.00)	1.03	(0.86, 1.22)

Marital status and educational levels showed no significant impact in the adjusted model. Occupational status before the pandemic and occupation changes after the first pandemic wave were also not significant factors in the adjusted analysis.

Individual income levels were not significant for testing rates in the adjusted model, however, higher household income maintained its significance in the adjusted model, indicating a continued relationship with increased testing rates. Larger household sizes did not show a significant difference in adjusted testing rates. Lower frequencies of TV watching and mobile internet usage were associated with lower adjusted PRs for testing. Using private healthcare facilities was associated with a slightly lower adjusted PR for testing compared to government facilities. Lastly, tobacco use in the last three years did not significantly influence testing in the adjusted model.

## Discussion

Our study shows insights into the determinants of SARS-CoV-2 testing in acute conditions among diverse socio-economic groups in Lucknow and Sitapur, two districts with distinctive demographic profiles. One of the findings from this study is the geographical variation in testing rates, with residents of Sitapur being tested significantly less than those in Lucknow, even after controlling for other variables. It was also evident from the fact that Sitapur has the lowest TPM while Lucknow reported the maximum TPM in the state and it was also the criteria for selecting these districts. This discrepancy might reflect inherent differences in health infrastructure, availability of testing centers, or public health policy implementation between the two districts.

Our adjusted models especially revealed that caste dynamics played a role in testing rates, with Scheduled Tribes showing a significantly higher likelihood of being tested compared to the General caste. This is contrary to much of the existing literature that typically cites lower healthcare utilization among Scheduled Tribes due to systemic barriers [22]. One possible explanation for our findings could be proactive measures taken to prioritize marginalized communities during the pandemic, an area that merits further exploration.

Higher household income was consistently associated with increased testing rates. This aligns with international research, suggesting that wealthier individuals are more likely to seek healthcare services due to better knowledge and more resources [23]. Our study reaffirms the importance of socio-economic status as a determinant of health-seeking behavior in the context of a global pandemic.

Our analysis points to an interesting correlation between media use and testing rates. Individuals who reported watching TV or using the internet less frequently had lower testing rates, suggesting that information dissemination through these media might influence health-seeking behaviors, as was also seen in the H1N1 pandemic response [24].

Preference for private healthcare facilities was associated with a lower testing rate, an unexpected finding considering the perceived quality and efficiency of private healthcare. This could imply potential barriers to testing within private settings or reflect different health-seeking behaviors among those who typically opt for private care.

Study limitations

The study presents valuable insights into the socioeconomic and demographic determinants of COVID-19 testing rates in UP. However, its findings must be interpreted within the context of inherent limitations. The exclusion of deceased individuals due to COVID-19 introduces survivorship bias, likely leading to an underrepresentation of the testing needs and barriers among the most vulnerable populations. Additionally, the potential unmeasured confounders such as mobility and exposure risk, and socio-cultural norms and beliefs around illness, healthcare seeking, and trust in the health system could significantly influence testing behaviors, suggesting that the observed associations might not fully capture the complex interplay of factors affecting testing uptake.

Moreover, the reliance on self-reported data could introduce recall bias, particularly in a rapidly evolving pandemic context where dates and symptoms might be challenging to remember accurately. This could lead to misclassification of testing status or symptoms, potentially biasing the results towards non-differential misclassification and diluting the true associations.

The cross-sectional design limits the ability to infer causality between the identified factors and testing behaviors. Longitudinal studies would be necessary to understand how these relationships evolve throughout the pandemic and to identify causal pathways.

## Conclusions

The identified predictors of SARS-CoV-2 testing - geographical location, caste, household income, and media use - underscore the need for customized public health strategies. Efforts to reduce disparities in testing rates should consider enhancing access in areas with limited healthcare infrastructure, employing targeted media campaigns to disseminate health information, and addressing potential barriers within private healthcare settings. Policymakers should also consider the nuanced influence of caste to ensure equitable healthcare delivery. Further longitudinal research is warranted to understand the dynamics of these relationships over time and across different stages of the pandemic. This study contributes to the growing body of evidence on health disparities during the COVID-19 pandemic and highlights the complexity of factors influencing testing rates. It underscores the need for multifaceted public health interventions that are sensitive to the socio-economic and cultural contexts of diverse populations.
